# Rice Germ Attenuates Chronic Unpredictable Mild Stress-Induced Muscle Atrophy

**DOI:** 10.3390/nu15122719

**Published:** 2023-06-12

**Authors:** Sosorburam Batsukh, Seyeon Oh, Kyoungmin Rheu, Bae-Jin Lee, Chang Hu Choi, Kuk Hui Son, Kyunghee Byun

**Affiliations:** 1 Department of Anatomy & Cell Biology, College of Medicine, Gachon University, Incheon 21936, Republic of Korea; sosorburam72@gmail.com; 2 Functional Cellular Networks Laboratory, Lee Gil Ya Cancer and Diabetes Institute, Gachon University, Incheon 21999, Republic of Korea; seyeon8965@gmail.com; 3 Marine Bioprocess Co., Ltd., Smart Marine BioCenter, Busan 46048, Republic of Korea; kmin.rheu@gmail.com (K.R.); hansola82@hanmail.net (B.-J.L.); 4 Department of Thoracic and Cardiovascular Surgery, Gil Medical Center, Gachon University, Incheon 21565, Republic of Korea; cch624@gilhospital.com; 5 Department of Health Sciences and Technology, Gachon Advanced Institute for Health & Sciences and Technology (GAIHST), Gachon University, Incheon 21999, Republic of Korea

**Keywords:** rice germ, gamma-aminobutyric acid, chronic unpredictable mild stress, muscle atrophy, cortisol

## Abstract

Chronic stress leads to hypothalamic–pituitary–adrenal axis dysfunction, increasing cortisol levels. Glucocorticoids (GCs) promote muscle degradation and inhibit muscle synthesis, eventually causing muscle atrophy. In this study, we aimed to evaluate whether rice germ supplemented with 30% γ-aminobutyric acid (RG) attenuates muscle atrophy in an animal model of chronic unpredictable mild stress (CUMS). We observed that CUMS raised the adrenal gland weight and serum adrenocorticotropic hormone (ACTH) and cortisol levels, and these effects were reversed by RG. CUMS also enhanced the expression of the GC receptor (GR) and GC–GR binding in the gastrocnemius muscle, which were attenuated by RG. The expression levels of muscle degradation-related signaling pathways, such as the Klf15, Redd-1, FoxO3a, Atrogin-1, and MuRF1 pathways, were enhanced by CUMS and attenuated by RG. Muscle synthesis-related signaling pathways, such as the IGF-1/AKT/mTOR/s6k/4E-BP1 pathway, were reduced by CUMS and enhanced by RG. Moreover, CUMS raised oxidative stress by enhancing the levels of iNOS and acetylated p53, which are involved in cell cycle arrest, whereas RG attenuated both iNOS and acetylated p53 levels. Cell proliferation in the gastrocnemius muscle was reduced by CUMS and enhanced by RG. The muscle weight, muscle fiber cross-sectional area, and grip strength were reduced by CUMS and enhanced by RG. Therefore, RG attenuated ACTH levels and cortisol-related muscle atrophy in CUMS animals.

## 1. Introduction

Chronic stress increases the levels of various hormones involved in the hypothalamic–pituitary–adrenal (HPA) axis, such as corticotropin-releasing hormone (CRH) from the hypothalamus, adrenocorticotropic hormone (ACTH) from the pituitary, and glucocorticoid (GC) from the adrenal cortex [1,2]. Increased CRH levels stimulate ACTH secretion, which eventually induces the adrenal glands to synthesize cortisol [3]. The over-secretion of these factors leads to HPA dysfunction and causes various disorders, such as depression [4,5,6]. Because the HPA axis plays essential roles in controlling body homeostasis in response to various stimuli, its dysfunction results in physiological and psychological abnormalities [7,8,9].

Chronic stress leads to muscle atrophy via HPA axis dysfunction [10,11]. Acute stress increases GC secretion, which further inhibits the HPA axis via a negative feedback mechanism [12,13,14]. However, chronic stress causes a dysfunction in this negative feedback mechanism, resulting in the persistent expression of GC [14]. By binding to the GC receptor (GR), GC increases the transcription of various target genes, such as the KLF transcription factor 15 (KLF15) and DNA damage inducible transcript 4 (DDIT4, also known as REDD-1), which are involved in skeletal muscle degradation [15]. KLF15 and REDD-1 also inhibit the mechanistic target of rapamycin kinase (mTOR) and lead to muscle atrophy [16,17].

The insulin-like growth factor (IGF)–protein kinase B (AKT)–mTOR pathway is the main pathway for the synthesis of muscle proteins via the activation of s6k1 and 4E-BP1 [18]. GC inhibits muscle synthesis, thus inhibiting those pathways [19,20,21]. IGF/AKT signaling decreases the expression levels of forkhead box O3 (FoxO3), atrogin-1, and muscle RING finger 1 (MuRF1), which are ubiquitin–proteasome protein degradation pathways [18]. Therefore, GC upregulates the expression levels of FoxO3, atrogin-1, and MuRF1 via the inhibition of the IGF/AKT pathway and increased muscle degradation [16,17]. GC also directly activates FoxO, leading to muscle degradation [22].

Increased cortisol or GR activity leads to the upregulation of inducible nitric oxide synthase (iNOS) expression, thereby increasing oxidative stress [23]. Oxidative stress is increased by the excessive synthesis of reactive oxygen species (ROS) or increased iNOS-derived NO expression [24]. ROS increase p53 activity via p53 acetylation and lead to cellular senescence by enhancing cell cycle arrest [25]. Upon nerve denervation, ROS increase the activity of p53 and cellular senescence, leading to muscle atrophy [24].

As GCs cause muscle atrophy via various signaling pathways, chronic stress, which increases GC levels, can also cause muscle atrophy. Various stresses, such as a combination of acoustic stress, restrain stress, and cage-switching stress, can decrease the muscle mass or cause muscle atrophy [26,27,28].

We previously reported that gamma-aminobutyric acid (GABA)-enriched rice germ (RG) decreases chronic unpredictable mild stress (CUMS)-induced depressive-like behavior by decreasing hypothalamic inflammation [29]. As RG decreases hypothalamic inflammation, it can also affect the HPA axis and modulate cortisol levels. Thus, we hypothesized that RG reduced CUMS-induced cortisol secretion, eventually attenuating GC-induced muscle atrophy. To confirm this, we evaluated whether CUMS induced muscle atrophy in this study. We also evaluated whether RG attenuates muscle atrophy by reducing Klf15 and Redd-1 levels and upregulating the IGF-1/AKT pathway. We found that RG lessened the iNOS and acetylated p53 levels, eventually attenuating muscle atrophy in CUMS animals.

## 2. Materials and Methods

### 2.1. RG Preparation

RG was prepared with reference to a previous study [29]. Briefly, the washed rice germ (1:10 ratio with water) was sprinkled with 1% amylase (Ban^®^ 480 L, FG, Novozymes, Seoul, Republic of Korea), and hydrolysis took place at a temperature of 67 ± 2 °C for 4 h. The hydrolyzed rice eyes were filtered with filter press (Daehan filter, Chung ju, Republic of Korea) and concentrated with Rotavapor (Merck KGaA, Darmstradt, Germany). 

A sterilized seed medium (3% yeast extract (Choheung, Ansan, Republic of Korea); 1% glucose (Qone, Seoul, Republic of Korea); 1% monosodium glutamate (CJ CheilJedang, Seoul, Republic of Korea); 95% water) was inoculated with *Lactobacillus brevis BJ20* (accession number KCTC 11377BP) and incubated at 37 °C for 24 h. Then, 10% (*v*/*v*) of the seed medium with *Lactobacillus brevis BJ20* was fermented in a fermentation medium (1.5% yeast extract (Choheung); 0.5% glucose (Qone); 8% monosodium glutamate (CJ CheilJedang); 24% L-glutamic acid (Samin chemical, Siheung, Republic of Korea); 50% hydrolyzed rice germ extract; 16% water) at 37 °C for 72 h. The fermentation medium underwent filtration using a filter press (Daehan filter). Dextrin (MATSUTANI KOREA, Seoul, Republic of Korea) was subsequently added to the filtered medium. The resulting mixture was then subjected to spray drying to prepare rice germ powder samples. RG was confirmed via high-performance liquid chromatography analysis [29].

### 2.2. Induction of CUMS and Oral Administration of RG in Animals

This study was approved by the Animal Care and Use Committee of Gachon University (approval no. LCDI-2021-0131). Male C57BL/6N mice (8-week-old) were purchased from Orient Bio (Seongnam, Republic of Korea). The mice were raised at approximately 23 °C and 50% relative humidity in a 12/12 h dark/light cycle.

After one week of adaptation, stress was induced for five weeks. The following stress induction procedures were applied: food divestment for 24 h, no water bottle in the mouse house for 24 h, and placing 200 mL of water in the mouse cage for 24 h. The mice subjected to stress were randomly allocated into six groups, with each group consisting of five mice. The control group was left unstressed for five weeks [29,30,31,32]. After five weeks of CUMS, mice were orally administered with saline, three concentrations of RG, or GABA daily for four weeks, and the CUMS animal model underwent a stress procedure during oral administration.

(1) Non-CUMS/Saline: Oral administration of saline at the same volume as the other group without stress.
(2) CUMS/Saline: Oral administration of saline at the same volume as the other group with stress.
(3) CUMS/RG 40: Oral administration of 40 mg/kg/day RG in saline with stress.
(4) CUMS/RG 90: Oral administration of 90 mg/kg/day RG in saline with stress.
(5) CUMS/RG 140: Oral administration of 140 mg/kg/day RG in saline with stress.
(6) CUMS/GABA: Oral administration of 30 mg/kg/day GABA in saline with stress.

### 2.3. Grip Strength

After four weeks of oral administration, a grip strength meter was utilized to assess the mice’s grip strength (JD-A-22; JEUNGDO BIO& PLANT Co., Ltd., Seoul, Republic of Korea). The mice were placed on a metal grid. After undergoing an adaptation process, their tails were smoothly tugged during measurements. Ten measurements were averaged for each mouse, and all measurements were performed within 5 min [33].

### 2.4. Sample Collection

After four weeks of oral administration, the mice were euthanized under respiratory anesthesia induced by 0.3% isoflurane (HANA Pharm Co., Ltd., Seoul, Republic of Korea) and 1.5% O_2_. Then, the spleen, adrenal gland, and blood samples were harvested to determine the stress modulating effect of RG, and gastrocnemius muscle was collected to confirm the effect of reducing stress-induced muscle atrophy [34]. The weight of the spleen, adrenal gland, and gastrocnemius muscle was measured. The longest transverse length of the gastrocnemius muscle was determined using a digital caliper (Mitoyo, Kanagawa, Japan).

### 2.5. Sample Preparation

#### 2.5.1. Serum Separation

In order to measure ACTH and the cortisol of the serum, a 1 mL aliquot of the collected blood was incubated in serum separator tubes (Becton Dickinson, Franklin Lakes, NJ, USA) for 20 min at room temperature. Subsequently, the blood specimen was subjected to centrifugation at 2000× *g* for 20 min at room temperature, and the supernatant was transferred into a new tube. 

#### 2.5.2. Protein Isolation

Gastrocnemius muscle tissues of five mice (20 mg per mouse) were pooled and homogenized using a glass tissue grinder (Wheaton Industries, Milville, NJ, USA) in 1 mL of EzRIPA buffer containing a protease inhibitor and phosphatase inhibitor (ATTO Corporation, Tokyo, Japan). Subsequently, the homogenized gastrocnemius muscle was incubated on ice for 15 min to facilitate cell lysis. Following sonication (high power, resting time 1 min, working time 10 s; CosmoBioCo., Ltd., Tokyo, Japan), the samples were centrifuged at 14,000× *g* for 15 min at 4 °C. Subsequently, the supernatants were collected, and the protein concentration was determined using a bicinchoninic acid assay kit (Thermo Fisher Scientific, Rockford, IL, USA), following the manufacturer’s instructions. 

#### 2.5.3. RNA Extraction and cDNA Synthesis

The gastrocnemius muscle tissues of five mice (10 mg per mouse) were pooled and lysed using a glass tissue grinder (Milville) in 1 mL of RNAiso reagent (TAKARA, Tokyo, Japan) for RNA extraction. After lysis, the samples were combined with 200 µL of chloroform (Samchun, Seoul, Republic of Korea) and subjected to centrifugation at 12,000× *g* for 15 min at 4 °C. This centrifugation step facilitated the separation of the RNA-containing aqueous phase from the rest of the component. The aqueous phase containing the RNA was carefully transferred to a new tube. To precipitate the RNA, 500 µL of isopropanol (Duksan, Seoul, Republic of Korea) was added and allowed to incubate for 10 min at room temperature. Subsequently, the RNA was pelleted by centrifugation at 12,000× *g* for 10 min at 4 °C. The resulting pellet was then washed with 1 mL of 75% cold ethanol (Sigma-Aldrich, St. Louis, MO, USA). The RNA pellet was air-dried for 10 min at room temperature and subsequently reconstituted in diethyl-pyrocarbonate-treated water (DEPC water; Biosesang, Seongnam, Republic of Korea). The purity and concentration of the RNA were determined using a NanoDrop spectrophotometer (Thermo Fisher Scientific).

To initiate cDNA synthesis, 1 µg of the extracted RNA was combined with Oligo DT primers (TAKARA) and dNTPs (TAKARA) in RNase-free distilled water (TAKARA), followed by a 5 min boiling step at 65 °C. Subsequently, the mixture was supplemented with reverse transcriptase (TAKARA), RNase inhibitor was further incubated in a thermal cycler (Bio-Rad Laboratories, Hercules, CA, USA) at 42 °C for 45 min. Finally, a final denaturation step was performed at 95 °C for 5 min to complete the cDNA synthesis process.

#### 2.5.4. Paraffin-Embedded Gastrocnemius Muscle Blocks

The gastrocnemius tissues were fixed in cold 4% paraformaldehyde (Sigma-Aldrich) for 72 h at 4 °C. Following fixation, the tissues were washed with tap water for 1 h and subsequently dehydrated in series of increasing ethanol concentrations. To facilitate transparency, the tissues were then cleared in xylene (Duksan) before being embedded in paraffin using a tissue processor (Leica, Wetzlar, Germany). The paraffin-embedded tissue blocks were then sectioned into 7 μm slices using a microtome (Thermo Fisher Scientific). The sections were placed onto coated microscope slides (Muto pure chemical Co., Ltd., Tokyo, Japan) and subjected to baking at 60 °C for 24 h to improve tissue adhesion.

### 2.6. Indirect-Enzyme-Linked Immunosorbent Assay (Indirect-ELISA)

The amounts of ACTH and cortisol were measured in the serum samples described in 2.5.1 via an indirect-ELISA. Briefly, 96-well microplates (LPS solution, Daejeon, Republic of Korea) were coated with a buffer (0.3% sodium carbonate (Sigma-Aldrich), 0.6% sodium bicarbonate (Sigma-Aldrich), and 91.9% distilled water) and left overnight at 4 °C. The coated microplates were washed with phosphate-buffered saline containing 0.1% Tween-20 (TPBS; LPS solution) and blocked with 5% skim milk (LPS solution) for 6 h at room temperature. After being washed with TPBS, 100 μg of serum was added into each well and then incubated overnight at 4 °C. After washing with TPBS, the microplates were incubated with anti-ACTH or anti-cortisol antibodies overnight at 4 °C (Table S1). Following washing with TBPS, the primary antibodies were incubated with a peroxidase-conjugated secondary antibody (Vector Laboratories, Burlingame, CA, USA) at room temperature for 4 h. After another round of washing, a tetramethylbenzidine solution (Sigma-Aldrich) was added and allowed to incubate in the dark at room temperature for 15–20 min to enable color development. To terminate the reaction, an equal volume of 2 N sulfuric acid (Sigma-Aldrich) was added. The optical density at 450 nm was measured using a microplate reader (Multiskan SkyHigh Photometer; Thermo Fisher Scientific).

### 2.7. Sandwich-ELISA 

Cortisol and GR binding were measured in the gastrocnemius muscle protein samples. First, 96-well microplates were coated with the anti-cortisol diluted in the coating buffer described in Section 2.6 (Table S1) and then incubated overnight at 4 °C. After washing with TPBS, they were blocked with 5% skim milk (Sigma-Aldrich) for 6 h at room temperature, and 60 μg of the isolated protein, as described in Section 2.5.2, was loaded into each well and incubated overnight at 4 °C. The microplates were washed with TPBS and incubated with anti-GR antibody overnight at 4 °C (Table S1). After washing again with TPBS, the microplates were incubated with a peroxidase-conjugated secondary antibody (Vector Laboratories) for 4 h at room temperature. Following the last TPBS wash, a tetramethylbenzidine solution (Sigma-Aldrich) was added and allowed to incubate for 20 min at room temperature. Then, an equal volume of a stop solution of 2 N sulfuric acid (Sigma-Aldrich) was added, and the optical density at 450 nm was determined using a microplate reader (Multiskan SkyHigh Photometer; Thermo Fisher Scientific).

### 2.8. Western Blotting

In total, 50 µg of the isolated protein, as described in Section 2.5.2, was mixed with 4× LDS sample buffer (Thermo Fisher Scientific), 10× sample reducing agent (Thermo Fisher Scientific), and distilled water. Subsequently, the mixed samples were subjected to denaturation at 70 °C for 10 min, followed by a 10 min cooling period on ice. Then, 3–8% Tris-Acetate gel (Invitrogen, Rockford, IL, USA) was used to verify the protein expression of mTOR and pmTOR, and 8% sodium dodecyl sulfate (SDS)-polyacrylamide gel was used to verify the protein expression of iNOS, FoxO3a, and pFoxO3a. In addition, 10% SDS-polyacrylamide gel was used to confirm the expression of other proteins. The denatured protein was electrophoresed at 200 V using a Tris-Acetate SDS running buffer (Invitrogen) for 3–8% Tris-Acetate gel (Invitrogen) and a MOPS buffer (Invitrogen) for 8% or 10% SDS-polyacrylamide gel. Next, the protein was transferred onto the polyvinylidene fluoride (PVDF) membranes (Merck Millipore, Burlington, MA, USA) using an electro-transferred Semi-dry transfer system (ATTO Corporation) with a current of 1 A for 10 min. Following the transfer, the membranes were blocked at room temperature for 1 h using a solution of 5% skim milk (LPS solution) in tris-buffered saline with 0.1% Tween 20 (TTBS; LPS solution). Following washing with TTBS, the primary antibody was appropriately diluted with TTBS according to the proportions specified in Table S1. The membranes were then incubated overnight at 4 °C with the primary antibody. Subsequently, the membranes were washed with TTBS and incubated with a horseradish peroxidase (HRP)-conjugated secondary antibody (Vector Laboratories) for 2 h at room temperature. Following the incubation, the membranes were washed with TTBS, and the protein bands were visualized using the ChemiDoc Imaging Systems (Bio-Rad Laboratories). This was achieved by exposing the membranes to an enhanced chemiluminescence solution (Cytiva^TM^, Marlborough, MA, USA) for a 3 min reaction. All protein bands were subsequently quantified using the ImageJ software (National Institutes of Health, NIH, Bethesda, MD, USA). The expression levels of β-actin were utilized as the internal control, and the fold change relative to the non-CUMS/saline group was determined and represented in each graph.

### 2.9. Quantitative Reverse Transcription–Polymerase Chain Reaction (qRT-PCR)

For qRT-PCR analysis, a total volume of 10 µL was prepared, consisting of 2.5 µL of cDNA template, 5 µL of ROX plus SYBR green premix (TAKARA), 0.8 µL of each reverse and forward primer (Table S2), and 0.9 µL of distilled water, as described in Section 2.5.3. The qRT-PCR amplification and melting curve analyses were carried out using a real-time PCR instrument (Thermo Fisher Scientific). The qRT-PCR protocol involved an initial denaturation step at 95 °C for 10 min, followed by 40 cycles of amplification consisting of denaturation at 95 °C for 15 s, annealing at 60 °C for 1 min, and extension at 95 °C for 15 s. After amplification, a melting analysis was performed from 60 °C to 95 °C at a rate of an increase of 0.075 °C/s. The gene expression levels were determined using the comparative CT method (ΔΔCT) [35]. The mRNA level was normalized to *Actb* and expressed relative to the level in the non-CUMS/saline group.

### 2.10. 3,3′-Diaminobenzidine (DAB) Staining

The tissue sections prepared as described in Section 2.5.4. were deparaffinized and rehydrated. This process involved sequential incubation in a series of xylene (Duksan) and a gradient of 100–70% alcohols (Duksan). The tissue sections were boiled in a sodium citrate buffer (pH 6.0) using a microwave oven for 20 s and cooled in distilled water for antigen retrieval. After washing with PBS, the tissue sections were incubated with a 1% bovine serum solution for 10 min at room temperature to block non-specific binding, followed by incubation with anti-PCNA antibody (Table S1) overnight at 4 °C. After washing with PBS, the slides were incubated with a biotinylated secondary antibody (Vector Laboratories) for 1 h at room temperature. The slides were incubated with an ABC kit (Vector Laboratories) for 30 min. The washed tissue sections were developed with a DAB solution (Sigma-Aldrich) for 15 min to obtain a brown color. For counterstaining, the tissue sections were incubated with hematoxylin (Korea pathology technical center, Cheong ju, Republic of Korea) for 30 s, washed with distilled water, dehydrated using graded alcohols (70–100%) and xylene, and mounted using a DPX mounting solution (Sigma-Aldrich). The stained tissues were imaged using a slide scanner (Motic Scan Infinity 100; Motic, Beijing, China). The number of PCNA-positive signals was counted per 50 µm^2^ using the ImageJ software (NIH).

### 2.11. Hematoxylin and Eosin (H&E) Staining

For the measurement of the gastrocnemius cross-sectional area (CSA), the gastrocnemius tissues were subjected to hematoxylin and eosin staining. Initially, the tissue sections were deparaffinized using xylene (Duksan) and subsequently rehydrated through a series of graded alcohols (100–70%). Following this, the sections were stained with hematoxylin solution (Korea pathology technical center) for 2 min and then washed with distilled water for 3 min. Next, the tissue sections were treated with 0.08% ammonia water (Korea Pathology Technical Center) for 30 s and washed in distilled water for 30 s and 95% alcohol for 30 s. The sections were then incubated with eosin solution (Korea pathology technical center) for 30 s, followed by a 3 min wash in distilled water. Subsequently, the sections underwent dehydration using a series of graded alcohols (70–100%), followed by being cleared in xylene (Duksan). Finally, the sections were mounted with a coverslip using a mounting medium (DPX solution; Sigma-Aldrich). The stained tissues were captured using a slide scanner (Motic Scan Infinity 100) to generate images. For each sample, the CSA of the gastrocnemius muscle was measured at 10 images. The obtained images were then subjected to analysis using the ImageJ software (NIH). 

### 2.12. Statistical Analysis

Statistical significance was determined using a one-way analysis of variance (ANOVA) test, followed by a post hoc Tukey’s test to compare each group. The data were presented as the mean ± standard error, pairwise comparisons were made between group means, and adjusted *p*-values were obtained. Significant differences were determined by comparing the adjusted *p*-values to the significance level. All statistical analyses were performed using SPSS version 22 (IBM Corporation, Armonk, NY, USA).

*, Non-CUMS/Saline vs. CUMS/Saline
$, CUMS/Saline vs. CUMS/RG or GABA
#, CUMS/RG vs. CUMS/GABA

## 3. Results

### 3.1. RG Attenuated ACTH and Cortisol Levels

RG contained 30% of GABA and 1% of lactic acid (Table S3). 

**Figure 1:**
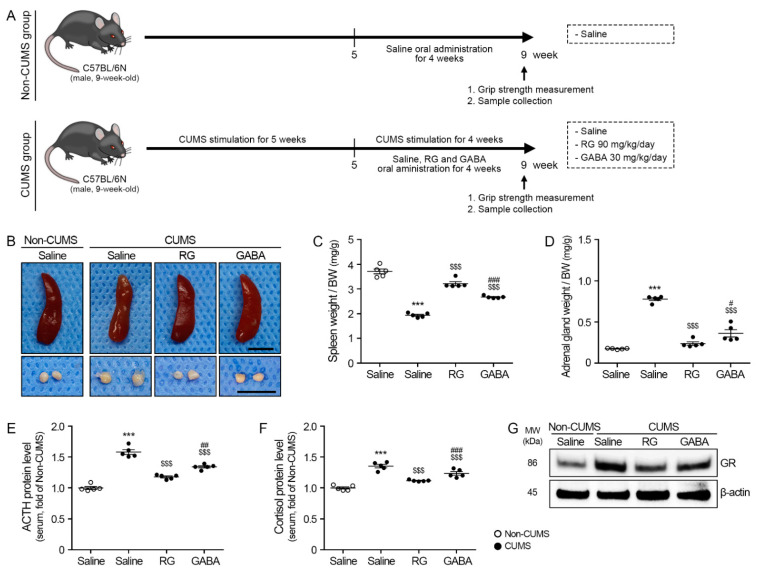
Rice germ (RG) decreases the adrenocorticotropic hormone (ACTH) and cortisol levels, which are increased by stress. (**A**) Schematic diagram of chronic unpredictable mild stress (CUMS)-induced animal experimental design. The CUMS procedure was conducted for 5 weeks. After the CUMS procedure for 5 weeks, RG (90 mg/kg/day) and GABA (30 mg/kg/day) were administered orally at the same time as the CUMS procedure for 4 weeks. After 4 weeks of oral administration, grip strength was performed, and samples (Serum, spleen, adrenal gland, and gastrocnemius muscle) were collected. (**B**) These are representative images of the spleen (upper) and adrenal gland (lower). Scale bar = 500 μm. (**C**,**D**) The quantitative graphs of the spleen (**C**) and adrenal gland (**D**) weight were normalized to the body weight. (**E**,**F**) Protein levels of ACTH (**E**) and cortisol (**F**) in serum were measured using an enzyme-linked immunosorbent assay (ELISA). (**G**) Protein levels of glucocorticoid receptor (GR) in gastrocnemius muscles were measured via Western blotting. In order to account for variations in protein loading, the Western blotting quantification was adjusted by normalizing it with β-actin, which served as a control protein for loading. Data are represented as the mean ± standard error (Sample size, *n* = 5). ***, *p* < 0.001, non-CUMS/Saline vs. CUMS/Saline; $$$, *p* < 0.001, CUMS/Saline vs. CUMS/RG or GABA; #, *p* < 0.05, ##, *p* < 0.01 or ###, *p* < 0.001, CUMS/RG vs. CUMS/GABA (Tukey’s test).

The administration dosage of RG was determined by serum ACTH and the cortisol levels of the CUMS-applied animals. RG of 40, 90, and 140 mg/kg were administered to CUMS-applied animals. The ACTH and cortisol levels of the RG 90 mg/kg-treated group were significantly lower than those of the RG 40 mg/kg-treated group. However, the ACTH and cortisol levels of the RG 90 mg/kg-treated group were not significantly different from those of the 140 mg/kg of RG-treated group. Thus, RG 90 mg/kg was administered to animals for further experiments (Figure S1 and Figure 1A).

Under CUMS, saline (CUMS/saline group), RG of 90 mg/kg (CUMS/RG group), and GABA of 30 mg/kg (CUMS/GABA group) were administered to the animals. The non-CUMS group (non-CUMS/saline group) was administered with saline. 

The average body weights of the four groups at the end of the experiment were evaluated. The body weight of the non-CUMS/saline group (32.66 ± 1.33 g) was significantly higher than that of the CUMS/saline (28.12 ± 0.29 g), CUMS/RG (29.56 ± 1.27 g), or CUMS/GABA (28.98 ± 1.28 g) groups. However, there was no significant difference among the CUMS/saline, CUMS/RG, and CUMS/GABA groups (Figure S2).

We evaluated whether CUMS caused any changes in the spleen and adrenal glands. The spleen and adrenal gland weights were normalized using the body weight. There was a significant reduction in the mean spleen weight, which was normalized using the body weight of the CUMS group compared to the non-CUMS group (1.94 ± 0.08 to 3.71 ± 0.20 mg/g). The mean spleen weight was normalized using the body weight of the CUMS/RG (3.21 ± 0.16 mg/g) and CUMS/GABA (2.67 ± 0.05 mg/g) groups and was significantly higher than that of the CUMS/saline group (Figure 1B,C). The mean adrenal gland weight that was normalized using the body weight of the CUMS/saline group (0.78 ± 0.03 mg/g) was significantly higher than that of the non-CUMS/saline group (0.18 ± 0.01 mg/g). The mean adrenal gland weight, which was normalized using the body weight of the CUMS/RG (0.24 ± 0.04 mg/g) and CUMS/GABA (0.36 ± 0.29 mg/g) groups, was significantly lower than that of the CUMS/saline group (Figure 1B,D).

The serum ACTH levels and cortisol levels were normalized using each value of the non-CUMS/saline groups. The serum ACTH of the CUMS/saline groups (1.65 ± 0.03) was significantly higher than that of the non-CUMS/saline groups. The serum ACTH of the CUMS/RG (1.18 ± 0.03) and CUMS/GABA (1.34 ± 0.04) groups was significantly lower than that of the CUMS/saline group (Figure 1E). The serum cortisol of the CUMS/saline groups (1.48 ± 0.02) was significantly higher than that of the non-CUMS/saline groups. The serum cortisol of the CUMS/RG (1.14 ± 0.03) and CUMS/GABA (1.23 ± 0.06) groups was significantly lower than that of the CUMS/saline group (Figure 1F).

The GR expression in the gastrocnemius muscle of the CUMS/saline group (2.35 ± 0.12) was significantly higher than that of the non-CUMS/saline groups. The GR expression in the gastrocnemius muscle of the CUMS/RG (1.43 ± 0.06) and CUMS/GABA (1.74 ± 0.08) groups was significantly lower than that of the CUMS/saline group (Figure 1G and Figure S3).

### 3.2. RG Attenuated Klf15/Redd-1 and Promoted mTOR/s6k and 4E-BP1 Levels

**Figure 2:**
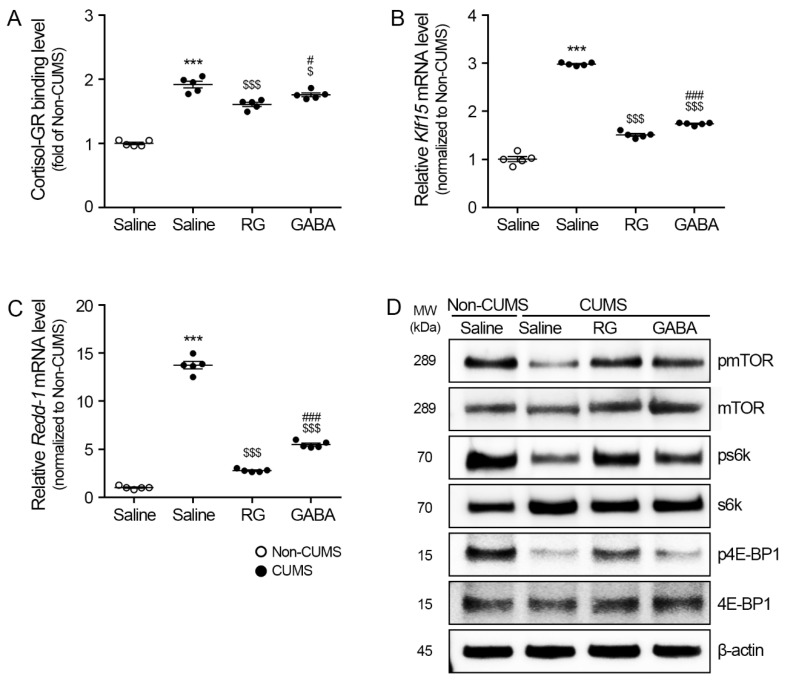
RG decreases the levels of the KLF transcription factor 15 (*Klf15*) and DNA damage inducible transcript 4 (Ddit4, also known as *Redd-1*) and increases the levels of the mechanistic target of rapamycin kinase (mTOR), s6K, and 4E-BP1. (**A**) Binding of cortisol to GR in the gastrocnemius muscle was measured via ELISA. (**B**,**C**) mRNA levels of *Klf15* (**B**) and *Redd-1* (**C**) in the gastrocnemius muscle were measured via quantitative reverse transcription–polymerase chain reaction (qRT–PCR). (**D**) Protein levels of mTOR, phosphorylated mTOR (pmTOR), s6k, ps6k, 4E-BP1, and p4E-BP1 in the gastrocnemius muscle were measured via Western blotting. In order to account for variations in protein loading, the Western blotting quantification was adjusted by normalizing it with β-actin, which served as a control protein for loading. To ensure consistency, the values for each blot were expressed relative to the average of the non-chronic unpredictable mild stress (CUMS) group. Data are represented as the mean ± standard error (Sample size, *n* = 5). ***, *p* < 0.001, non-CUMS/Saline vs. CUMS/Saline; $, *p* < 0.05 or $$$, *p* < 0.001, CUMS/Saline vs. CUMS/RG or GABA; #, *p* < 0.05 or ###, *p* < 0.001, CUMS/RG vs. CUMS/GABA (Tukey’s test).

The binding between cortisol and GR in the gastrocnemius muscle was evaluated using sandwich ELISA. The binding between the cortisol and GR of the CUMS/saline group (1.92 ± 0.10) was significantly higher than that of the non-CUMS/saline groups. The binding between the cortisol and GR of the CUMS/RG (1.61 ± 0.07) and CUMS/GABA (1.76 ± 0.06) groups was significantly lower than that of the CUMS/saline group (Figure 2A).

The mRNA expression levels of *Klf15* in the gastrocnemius muscle of the CUMS/saline group (2.98 ± 0.03) were significantly higher than those of the non-CUMS/saline groups. The mRNA expression levels of *Klf15* in the CUMS/RG (1.51 ± 0.06) and CUMS/GABA (1.74 ± 0.03) groups were significantly lower than those of the CUMS/saline group (Figure 2B).

The mRNA expression levels of *Redd-1* in the gastrocnemius muscle of the CUMS/saline group (13.75 ± 0.78) were significantly higher than those of the non-CUMS/saline groups. The mRNA expression levels of *Redd-1* in the CUMS/RG (2.77 ± 0.14) and CUMS/GABA (5.48 ± 0.31) groups were significantly lower than those of the CUMS/saline group (Figure 2C). 

The ratio of phosphorylated mTOR and mTOR (pmTOR/mTOR) in the gastrocnemius muscle of the CUMS/saline group (0.41 ± 0.07) was significantly lower than that of the non-CUMS/saline groups. The pmTOR/mTOR ratio of the CUMS/RG (0.76 ± 0.02) and CUMS/GABA (0.61 ± 0.01) groups was significantly higher than that of the CUMS/saline group (Figure 2D and Figure S4A).

The ratio of phosphorylated s6k and s6k (ps6k/s6k) in the gastrocnemius muscle of the CUMS/saline group (0.28 ± 0.07) was significantly lower than that of the non-CUMS/saline groups. The ps6k/s6k ratios of the CUMS/RG (0.61 ± 0.03) and CUMS/GABA (0.44 ± 0.06) groups were significantly higher than that of the CUMS/saline group (Figure 2D and Figure S4B).

The ratio of phosphorylated 4E-BP1 and 4E-BP1 (p4E-BP1/4E-BP1) in the gastrocnemius muscle of the CUMS/saline group (0.20 ± 0.02) was significantly lower than that of the non-CUMS/saline groups. The p4E-BP1/4E-BP1 ratio of the CUMS/RG (0.67 ± 0.04) and CUMS/GABA (0.35 ± 0.01) groups was significantly higher than that of the CUMS/saline group (Figure 2D and Figure S4C).

### 3.3. RG Promoted IGF-1/AKT and Attenuated FoxO3a, Atrogin-1, and MuRF1 Levels

**Figure 3:**
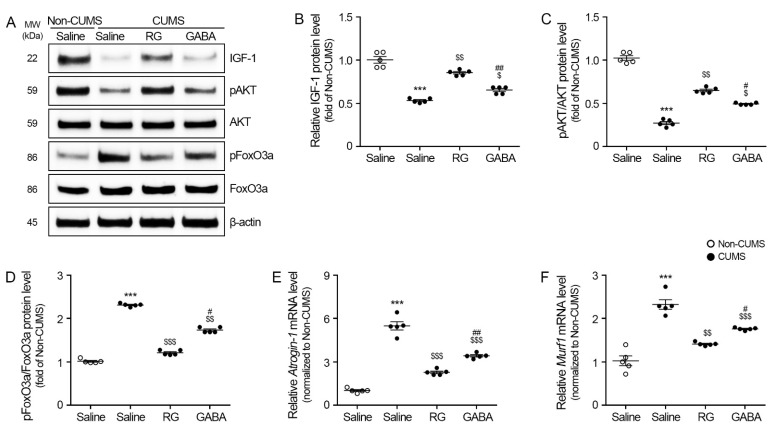
RG increases insulin-like growth factor 1 (IGF-1) and protein kinase B (AKT) levels and decreases forkhead box O3A (FoxO3a), *Atrogin-1*, and *Murf1* levels. (**A**) Protein levels of IGF-1, AKT, phosphorylated AKT (pAKT), FoxO3a, and phosphorylated FoxO3a (pFoxO3a) in the gastrocnemius muscle measured via Western blotting. (**B**–**D**) Quantitative graph for Western blotting of IGF-1 (**B**), pAKT/AKT (**C**), and pFoxO3a/FoxO3a (**D**). In order to account for variations in protein loading, the Western blotting quantification was adjusted by normalizing it with β-actin, which served as a control protein for loading. To ensure consistency, the values for each blot were expressed relative to the average of the non-chronic unpredictable mild stress (CUMS) group. (**E**,**F**) mRNA levels of *Atrogin-1* (**E**) and *Murf1* (**F**) in the gastrocnemius muscle were measured via qRT–PCR. Data are represented as the mean ± standard error (Sample size, *n* = 5). ***, *p* < 0.001, non-CUMS/Saline vs. CUMS/Saline; $, *p* < 0.05, $$, *p* < 0.01 or $$$, *p* < 0.001, CUMS/Saline vs. CUMS/RG or GABA; #, *p* < 0.05 or ##, *p* < 0.01, CUMS/RG vs. CUMS/GABA (Tukey’s test).

The IGF-1 level in the gastrocnemius muscle of the CUMS/saline group (0.10 ± 0.01) was significantly lower than that of the non-CUMS/saline groups. The IGF level of the CUMS/RG (0.59 ± 0.05) and CUMS/GABA (0.27 ± 0.010) groups was significantly higher than that of the CUMS/saline group (Figure 3A,B).

The ratio of phosphorylated AKT and AKT (pAKT/AKT) in the gastrocnemius muscle of the CUMS/saline group (0.37 ± 0.03) was significantly lower than that of the non-CUMS/saline groups. The pAKT/AKT ratio of the CUMS/RG (0.84 ± 0.04) and CUMS/GABA (0.50 ± 0.10) groups was significantly higher than that of the CUMS/saline group (Figure 3A,C).

The ratio of phosphorylated FoxO3a and FoxO3a (pFoxO3a/FoxO3a) in the gastrocnemius muscle of the CUMS/saline group (2.24 ± 0.17) was significantly higher than that of the non-CUMS/saline groups. The pFoxO3a/FoxO3a ratio of the CUMS/RG (1.14 ± 0.09) and CUMS/GABA (1.57 ± 0.10) groups was significantly lower than that of the CUMS/saline group (Figure 3A,D). 

*Atrogin-1* expression in the gastrocnemius muscle of the CUMS/saline group (5.48 ± 0.57) was significantly higher than that of the non-CUMS/saline groups. *Atrogin-1* expression in the CUMS/RG (2.27 ± 0.18) and CUMS/GABA (3.41 ± 0.16) groups was significantly lower than that of the CUMS/saline group (Figure 3E). 

*Murf1* expression in the gastrocnemius muscle of the CUMS/saline group (2.32 ± 0.22) was significantly higher than that of the non-CUMS/saline groups. The *Murf1* expression of the CUMS/RG (1.41 ± 0.04) and CUMS/GABA (1.76 ± 0.03) groups was significantly lower than that of the CUMS/saline group (Figure 3F). 

### 3.4. RG Attenuated iNOS/p53 and Promoted Cyclin-Dependent Kinase 2 (CDK2)/Cyclin D1 Levels

**Figure 4:**
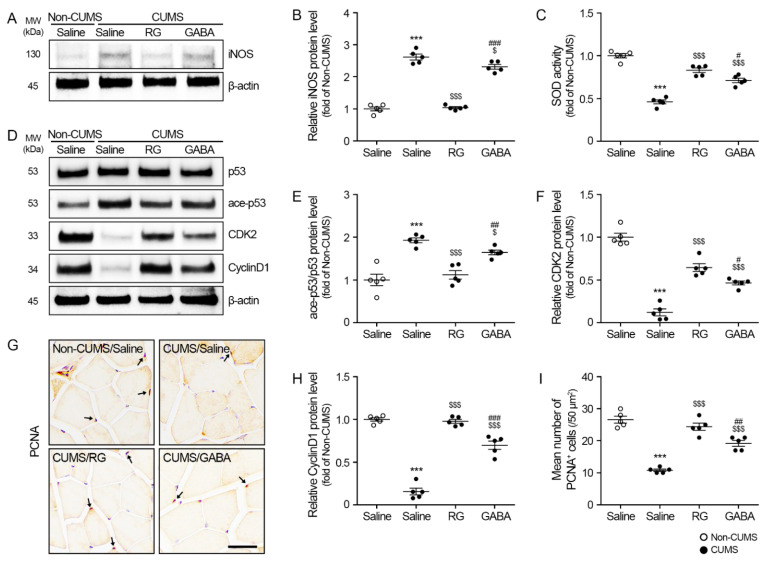
RG decreases inducible nitric oxide synthase (iNOS), reactive oxygen species (ROS), and p53 levels and increases cyclin-dependent kinase 2 (CDK2) and cyclin D1 levels. (**A**) iNOS expression levels in the gastrocnemius muscle measured via Western blotting. (**B**) Quantitative graph for Western blotting of (**A**). (**C**) SOD activity in the gastrocnemius muscle measured via ELISA. (**D**) Protein levels of p53, ace-p53, CDK2, and Cyclin D1 in the gastrocnemius muscle measured via Western blotting. (**E**,**F**,**H**) Quantitative graph for Western blots of ace-p53/p53 (**E**), CDK2 (**F**), and cyclin D1 (**H**) levels. To account for variations in protein loading, the Western blotting quantification was adjusted by normalizing it with β-actin, which served as a control protein for loading. To ensure consistency, the values for each blot were expressed relative to the average of the non-CUMS group. (**G**) Proliferating cell nuclear antigen (PCNA) expression levels in the gastrocnemius muscle measured via immunohistochemistry (The arrow points to the PCNA positive signal). Scale bar = 60 μm. (**I**) Quantitative graph for immunohistochemistry of (**G**). Data are represented as the mean ± standard error (Sample size, *n* = 5). ***, *p* < 0.001, non-CUMS/Saline vs. CUMS/Saline; $, *p* < 0.05, or $$$, *p* < 0.001, CUMS/Saline vs. CUMS/RG or GABA; #, *p* < 0.05, ##, *p* < 0.01 or ###, *p* < 0.001 CUMS/RG vs. CUMS/GABA (Tukey’s test).

The expression of iNOS in the gastrocnemius muscle of the CUMS/saline group (2.62 ± 0.18) was significantly higher than that of the non-CUMS/saline groups. The expression of iNOS in the CUMS/RG (1.04 ± 0.06) or CUMS/GABA (2.31 ± 0.15) groups was significantly lower than that of the CUMS/saline group (Figure 4A,B).

Superoxide dismutase (SOD) acts against the oxidative stress that catalyzes the conversion of superoxide anion free radicals into hydrogen peroxide and oxygen [36]. The SOD activity in the gastrocnemius muscle of the CUMS/saline group (0.46 ± 0.05) was significantly lower than that of the non-CUMS/saline groups. The SOD activity of the CUMS/RG (0.83 ± 0.05) and CUMS/GABA (0.71 ± 0.05) groups was significantly higher than that of the CUMS/saline group (Figure 4C). 

The ratio of acetylated p53-to-p53 (ace-p53/p53) in the gastrocnemius muscle of the CUMS/saline group (1.99 ± 0.06) was significantly higher than that of the non-CUMS/saline groups. The ace-p53/p53 ratio of the CUMS/RG (1.12 ± 0.20) and CUMS/GABA (1.61 ± 0.07) groups was significantly lower than that of the CUMS/saline group (Figure 4D,E).

Cell cycle arrest was evaluated by measuring the expression levels of CDK2 and cyclin D1. Cyclin D1 is involved in the cell cycle progression from G_0_/G_1_ to the S phase [37]. CDK2 also increases the entry of cells into the S phase [38]. The CDK2 expression in the gastrocnemius muscle of the CUMS/saline group (0.12 ± 0.08) was significantly lower than that of the non-CUMS/saline groups. The CDK2 expression of the CUMS/RG (0.64 ± 0.10) and CUMS/GABA (0.46 ± 0.05) groups was significantly higher than that of the CUMS/saline group (Figure 4D,F). The Cyclin D1 expression in the gastrocnemius muscle of the CUMS/saline group (0.16 ± 0.08) was significantly lower than that of the non-CUMS/saline groups. The Cyclin D1 expression of the CUMS/RG (0.98 ± 0.05) and CUMS/GABA (0.70 ± 0.10) groups was significantly higher than that of the CUMS/saline group (Figure 4D,H).

Cell proliferation was evaluated via proliferating cell nuclear antigen (PCNA) staining. The number of PCNA-positive cells in the gastrocnemius muscle of the CUMS/saline group (10.8 ± 0.75) was significantly lower than that of the non-CUMS/saline groups (26.6 ± 2.15). The number of PCNA-positive cells in the CUMS/RG (24.4 ± 2.24) and CUMS/GABA (19.2 ± 1.83) groups was significantly higher than that of the CUMS/saline group (Figure 4G,I).

### 3.5. RG Attenuates Muscle Atrophy in CUMS Mice

**Figure 5:**
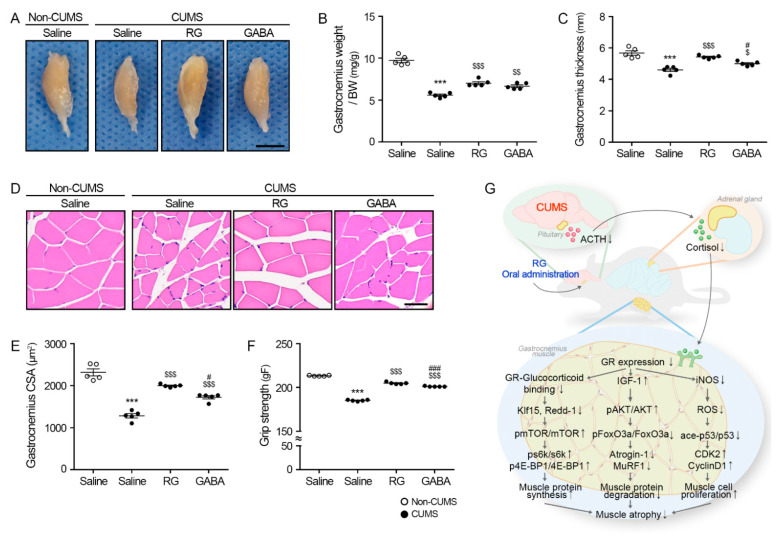
RG attenuates muscle atrophy caused by stress. (**A**) These are representative images of the gastrocnemius muscle. Scale bar = 5 mm. (**B**) A quantitative graph of gastrocnemius muscle weight was normalized to body weight. (**C**) This is a graph of and the longest transverse length of the gastrocnemius muscle. (**D**,**E**) The gastrocnemius muscle fiber cross-sectional area was measured via hematoxylin and eosin (H&E) staining. Scale bar = 30 μm. (**F**) Grip strength was measured using a meter. (**G**) Summary of this study. RG decreased (↓) stress hormone (ACTH and cortisol) secretion and downregulated (↓) the expression of GR, Klf15, and Redd-1, leading to the increased (↑) activation of the mTOR/s6k/4E-BP1 pathway. Additionally, RG elevated (↑) the IGF-1/AKT levels, decreased (↓) the FoxO3a/Atrogin-1/MuRF1 levels, and reduced (↓) the iNOS and acetylated p53 levels, resulting in enhanced (↑) expression levels of CDK2 and cyclin D1. Furthermore, RG improved (↑) the gastrocnemius muscle fiber CSA and grip strength in chronic unpredictable mild stress (CUMS) animals. Data are represented as the mean ± standard error (Sample size, *n* = 5). ***, *p* < 0.001, non-CUMS/Saline vs. CUMS/Saline; $, *p* < 0.05, $$, *p* < 0.01 or $$$, *p* < 0.001, CUMS/Saline vs. CUMS/RG or GABA; #, *p* < 0.05 or ###, *p* < 0.001, CUMS/RG vs. CUMS/GABA (Tukey’s test).

The weight of the gastrocnemius muscle, which was normalized using the body weight of the CUMS/saline group (5.59 ± 0.26 mg/g), was significantly lower than that of the non-CUMS/saline groups (9.73 ± 0.51 mg/g). The weight of the gastrocnemius muscle of the CUMS/RG (7.02 ± 0.36 mg/g) and CUMS/GABA (6.66 ± 0.32 mg/g) groups was significantly higher than that of the CUMS/saline group (Figure 5A,B).

The gastrocnemius muscle thickness (longest transverse length of the muscle) of the CUMS/saline group (4.61 ± 0.20 mm) was significantly lower than that of the non-CUMS/saline groups (5.68 ± 0.28 mm). The gastrocnemius muscle thickness of the CUMS/RG (5.43 ± 0.10 mm) and CUMS/GABA (5.00 ± 0.13 mm) groups was significantly higher than that of the CUMS/saline group (Figure 5A,C).

The muscle fiber CSA of the gastrocnemius muscle of the CUMS/saline group (1284.19 ± 103.33 μm^2^) was significantly lower than that of the non-CUMS/saline groups (2319.02 ± 167.41 μm^2^). The muscle fiber CSA of the gastrocnemius muscle of the CUMS/RG (2000 ± 31.42 μm^2^) and CUMS/GABA (1724.67 ± 78.84 μm^2^) groups was significantly higher than that of the CUMS/saline group (Figure 5D,E).

The grip strength of the CUMS/saline group (185.2 ± 0.75 gF) was significantly lower than that of the non-CUMS/saline groups (213.4 ± 0.49 gF). The grip strength of the CUMS/RG (205 ± 1.10 gF) and CUMS/GABA (201.2 ± 0.40 gF) groups was significantly higher than that of the CUMS/saline group (Figure 5F).

## 4. Discussion

GABA decreases the secretion of CRH, eventually decreasing cortisol secretion from the adrenal cortex [39,40]. Natural sources, such as fermented milk products, beans, brown rice sprouts, and barley, contain high amounts of GABA [41,42]. GABA-enriched rice has been reported to decrease anxiety and cortisol secretion [43]. Previously, our group reported that RG, which contains more than 30% GABA, decreases CUMS-induced neuroinflammation [29]. 

The CUMS animal model is a relevant model for evaluating stress-related depression or anxiety, as CUMS induces reproducible neuroendocrine changes and neuroinflammation [44,45,46,47,48,49,50]. CUMS also increases the serum cortisol levels [51,52]. GCs are known to cause muscle atrophy. Therefore, animal models injected with GCs, such as dexamethasone, have been widely used to evaluate muscle atrophy [40,41,42]. GC leads to greater atrophy in fast-twitch muscles, such as the gastrocnemius muscle, than in slow-twitch muscles, such as the soleus muscle [53,54]. GC injection decreases the muscle weight and myofiber CSA in the gastrocnemius muscle [55]. 

We evaluated the effects of CUMS on serum ACTH and cortisol levels in this study. Increased adrenal weight in stressed animals suggests the hyperactivation of the HPA axis [52]. In CUMS animals, the adrenal gland weight increases [56]. Similar to previous studies, our results showed that the adrenal gland weight of the CUMS/saline group was higher than that of the non-CUMS group. The adrenal gland weight of the RG- or GABA-administrated CUMS-applied groups was lower than that of the saline-administrated CUMS group. The serum ACTH and cortisol levels which were promoted by CUMS were attenuated via RG or GABA administration. 

GC leads to muscle atrophy by increasing muscle degradation via the upregulation of KLF15 and REDD-1 levels [15,16,17] and decreasing muscle synthesis via the downregulation of the IGF-1/AKT/mTOR/s6k/4E-BP1 levels [19,20,21]. In our study, CUMS promoted Klf15 and Redd-1 levels, which were attenuated by RG and GABA. CUMS also reduced the expression levels of mTOR/s6k/4E-BP1, which were promoted by RG and GABA. Moreover, CUMS promoted the levels of various muscle-depredating systems, such as FoxO3a, Atrogin-1, and MuRF1, in the muscle, and they were lessened by RG and GABA. 

CUMS induced changes in various GC-related signaling pathways involved in muscle degradation or muscle synthesis. In addition to its direct effect on these cells signaling pathways, GC increased the oxidative stress, thereby increasing the cellular senescence and muscle atrophy [24,25]. In our study, CUMS increased oxidative stress by promoting iNOS expression and reducing SOD activity, which were reversed by RG and GABA. Increased oxidative stress occurs during cancer, nerve denervation, and chronic diseases, such as diabetes and aging, eventually inducing muscle atrophy [57,58,59,60,61]. CUMS also upregulated the acetylation of p53 levels and enhanced cell cycle arrest, which aligned with the levels of cyclin D1 and CDK2. Those levels were attenuated by both RG and GABA. Increased p53 or oxidative stress in the muscle has been frequently reported as one of the main pathophysiologies of muscle atrophy. Increased ROS leads to p53 activity, which results in premature muscle atrophy [24]. Increased oxidative stress and p53 activity are shown in the muscle during aging [62]. Moreover, increased p53 was shown in the muscle atrophy of young animals in a state of limb immobilization [63]. It is known that the acetylation of p53 results in increased p53 stability, which upregulates transcriptional activity [64]. The acetylation of p53 results in cell cycle arrest or apoptosis [65,66]. Cell cycle arrest induces cellular senescent and satellite cell dysfunction, which lead to muscle atrophy [67]. 

PCNA is one of the nuclear nonhistone proteins and is required for DNA synthesis. Since PCNA is an accessory protein of DNA polymerase-α, it elevated during the G1/S phase [68]. Thus, the PCNA levels of senescent or quiescent cells are very low [68]. Moreover, PCNA levels are frequently used as markers of cell proliferation, since cells stay for longer in the G1/S phase when they are proliferated [69]. Our study results showed that the PCNA-positive cell number in the muscle was lower in the CUMS-applied animals group compared to that in the normal control group. RG and GABA enhanced the PCNA-positive cell number in the muscle. Those results suggested that CUMS enhanced oxidative stress and the acetylation of p53, which eventually led to promoted cell cycle arrest and lessened proliferation in the muscle. Those effects were attenuated by RG or GABA. 

GABA decreases high-fat-diet-induced oxidative stress in the gastrocnemius muscle, accompanied by increased SOD expression [70,71]. GABA also decreases oxidative stress in the gastrocnemius muscles of dexamethasone-treated animals [71]. GABA attenuates the dexamethasone-induced decrease in the CSA of muscle fibers and grip strength [71]. In our study, both GABA and RG promoted the gastrocnemius muscle weight and the CSA in CUMS animals. In addition, GABA and RG elevated the grip strength. The elevation in grip strength was greater in the RG group than in the GABA group. 

When animals consumed 90 mg/kg of RG, the amount of GABA consumed was approximately 27 mg/kg [29]. Thus, we administered 30 mg/kg of GABA to animals to compare the attenuation effect on muscle atrophy between RG and single treatment with GABA. In addition to GABA, rice germ contains various essential amino acids such as lysine, valine, and histidine [72]. Essential amino acids are the main components of muscle synthesis [73]. Moreover, RG contained 1% lactic acid. It is known that lactic acid stimulates muscle regeneration [74]. Therefore, the attenuating effect of RG on muscle atrophy was superior to that of GABA in CUMS animals. 

In this study, we did not evaluate the amount of food intake during the experiment. Since decreased food intake could induce nutritional deficiency, which affects muscle mass, the fact that food intake was not considered is a limitation of our study. However, the body weight of the CUMS-applied groups was not significantly different. RG or GABA did not significantly raise the body weight of animals under CUMS; however, muscle atrophy was attenuated by RG or GABA. Although the amount of the diet consumed by animals was not directly measured, it can be assumed that the change in food intake did not have a significant effect on muscle mass, since the body weight was similar in the three groups.

Muscle atrophy, characterized by the loss of muscle function and mass, is a serious health problem, especially in the elderly population. Muscle atrophy leads to increased disability, falls, fall-related injuries, and hospitalization [75]. It also increases functional decline and mortality [75]. As muscle atrophy decreases physical function, it negatively affects the quality of life [76]. In addition to aging, muscle atrophy is accompanied by various diseases, such as cancer, heart failure, chronic respiratory disease, sepsis, and infectious diseases [77,78]. Lifestyle factors, such as decreased physical activity and exercise, smoking, severe alcohol consumption, and a poor nutritional state, also cause muscle atrophy [79,80,81,82,83]. Various lifestyle modifications, such as increased exercise, low smoking, alcohol cessation, and increased protein consumption, have been suggested to decrease muscle atrophy [84].

Physical and psychological stressors affect the HPA axis [85,86]. Emotional stress can also lead to muscle atrophy [87,88,89], but it is difficult to manage emotional stress. Stress causes changes in the HPA axis, which results in a chronic increase in cortisol levels. 

In our study, RG lessened the cortisol levels and muscle atrophy, exerting a protective effect on muscle atrophy induced by various stresses. As emotional stress can lead to muscle atrophy, which aggravates other health conditions, RG may be used to treat patients under such emotional stress.

## 5. Conclusions

In conclusion, we found that RG attenuated the ACTH and cortisol secretion in CUMS animals in this study. Moreover, RG reduced the expression levels of GR, Klf15, and Redd-1, leading to the upregulation of the mTOR/s6k/4E-BP1 pathway. Additionally, RG raised the IGF-1/AKT levels and lessened the FoxO3a/Atrogin-1/MuRF1 levels. RG also lessened the iNOS and acetylated p53 levels, thereby enhancing the expression levels of CDK2 and cyclin D1. Lastly, RG raised the gastrocnemius muscle fiber CSA and grip strength in CUMS animals (Figure 5G). 

## Data Availability

Not applicable.

## Supplementary Materials

The following supporting information can be downloaded at: https://www.mdpi.com/article/10.3390/nu15122719/s1. Figure S1: Stress-induced increases in adrenocorticotropic hormone (ACTH) and cortisol levels in chronic unpredictable mild stress (CUMS) mice were reduced in a rice germ (RG) concentration-dependent manner; Figure S2: Rice germ (RG) does not cause body weight changes in chronic unpredictable mild stress (CUMS) mice; Figure S3: Rice germ (RG) decreases the glucocorticoid receptor (GR) in the gastrocnemius muscle of CUMS mice; Figure S4: Rice germ (RG) increases the pmTOR/mTOR, ps6k/s6k, and p4E-BP/4E-BP ratios in the gastrocnemius muscle of CUMS mice; Table S1: List of antibodies Western blotting, ELISA, and IHC; Table S2: List of primers of qRT-PCR; Table S3: RG contained 30% of GABA.
