# Supplementary material for: Rice Germ Attenuates Chronic Unpredictable Mild Stress-Induced Muscle Atrophy
Source: Nutrients. 2023 Jun 12;15(12):2719. doi: 10.3390/nu15122719 (PMC10303162; doi:10.3390/nu15122719)
Supplement: Supplementary file 1 [file nutrients-15-02719-s001.zip › nutrients-2446043-supplementary.pdf]

Supplementary materials and methods

1. High-Performance Liquid Chromatography (HPLC) Analysis

1.1. GABA HPLC analysis

GABA HPLC analysis was performed as described in the previous study [1]. Briefly, Dionex U3000 series equipped with a UV detector (Thermo Fisher Scientific, Rockford, IL, USA) was utilized for HPLC analysis to analyze the GABA content. Solvent A was composed of 50 mM sodium acetate (pH 6.5; Thermo Fisher Scientific), and solvent B consisted of 45% (v/v) acetonitrile (Thermo Fisher Scientific), 45% (v/v) methanol (Thermo Fisher Scientific), and 10% (v/v) distilled water. The flow rate was adjusted to 1 mL/min. The linear gradient was carried out over a period of 30 min at a wavelength of 338 nm, with an injection volume of 20 μL.

1.2. Lactic acid HPLC analysis

The content of lactic acid was determined using an HPLC system (Dionex U3000; Thermo Fisher Scientific) equipped with a UV detector. A 0.2 M phosphate butter solution (Potassium Phosphate, pH 2.4) was used as the mobile phase. Isocratic elution was conducted for 15 min at 214 nm, the injection volume was 20 μL, and the solvent flow rate was adjusted to 0.8 mL/min.

Supplementary figures

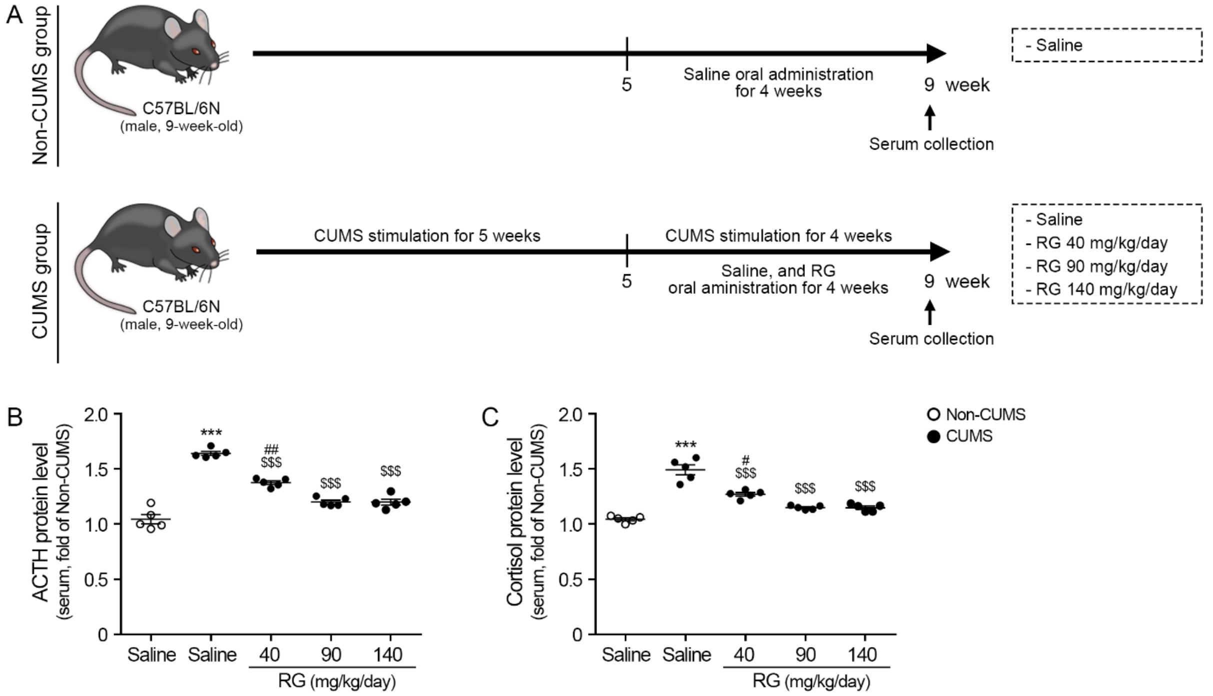

Figure S1. Stress-induced increases in adrenocorticotropic hormone (ACTH) and cortisol levels in chronic unpredictable mild stress (CUMS) mice were reduced in a rice germ (RG) concentration-dependent manner. (**A**) Schematic diagram of CUMS-induced animal experimental design. The CUMS procedure was conducted for 5 weeks. After the CUMS procedure for 5 weeks, RG (40, 90 and 140 mg/kg/day) was administered orally at the same time as the CUMS procedure for 4 weeks. After 4 weeks of oral administration, serum was collected. (**B and C**) Protein levels of ACTH (B) and cortisol (C) in serum were measured using an enzyme-linked immunosorbent assay (ELISA). Data are represented as the mean ± standard error of five independent experiments (Sample size, $n = 5$). ***, $p < 0.001$, Non-CUMS/Saline vs. CUMS/Saline; $$$, $p < 0.001$, CUMS/Saline vs. CUMS/RG or GABA; #, $p < 0.05$ or ##, $p < 0.01$ CUMS/RG vs. CUMS/GABA (Tukey's test).

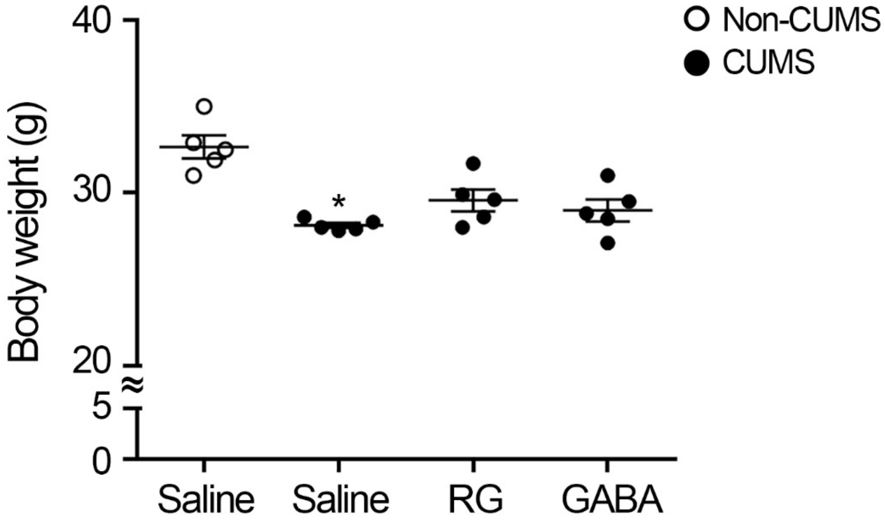

Figure S2. Rice germ (RG) does not cause body weight changes in chronic unpredictable mild stress (CUMS) mice. The CUMS mice were weighed after 4 weeks of oral administration of RG and GABA. Data are represented as the mean ± standard error of five independent experiments (Sample size, $n = 5$). *, $p < 0.05$, Non-CUMS/Saline vs. CUMS/Saline (Tukey's test).

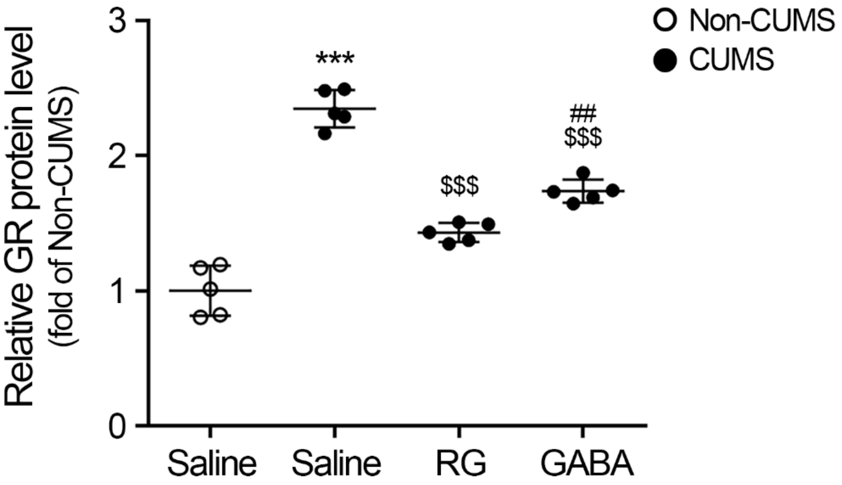

Figure S3. Rice germ (RG) decreases the glucocorticoid receptor (GR) in the gastrocnemius muscle of CUMS mice. (**A**) GR levels in gastrocnemius of CUMS mice were analyzed via Western blotting. Graph quantifying the data in Figure 1G. To account for variations in protein loading, the Western blotting quantification was adjusted by normalizing it with β-actin, which served as a control protein for loading. Data are represented as the mean ± standard error of five independent experiments (Sample size, $n = 5$). ***, $p < 0.001$, Non-CUMS/Saline vs. CUMS/Saline; $$$, $p < 0.001$, CUMS/Saline vs. CUMS/RG or GABA; ##, $p < 0.01$ CUMS/RG vs. CUMS/GABA (Tukey's test).

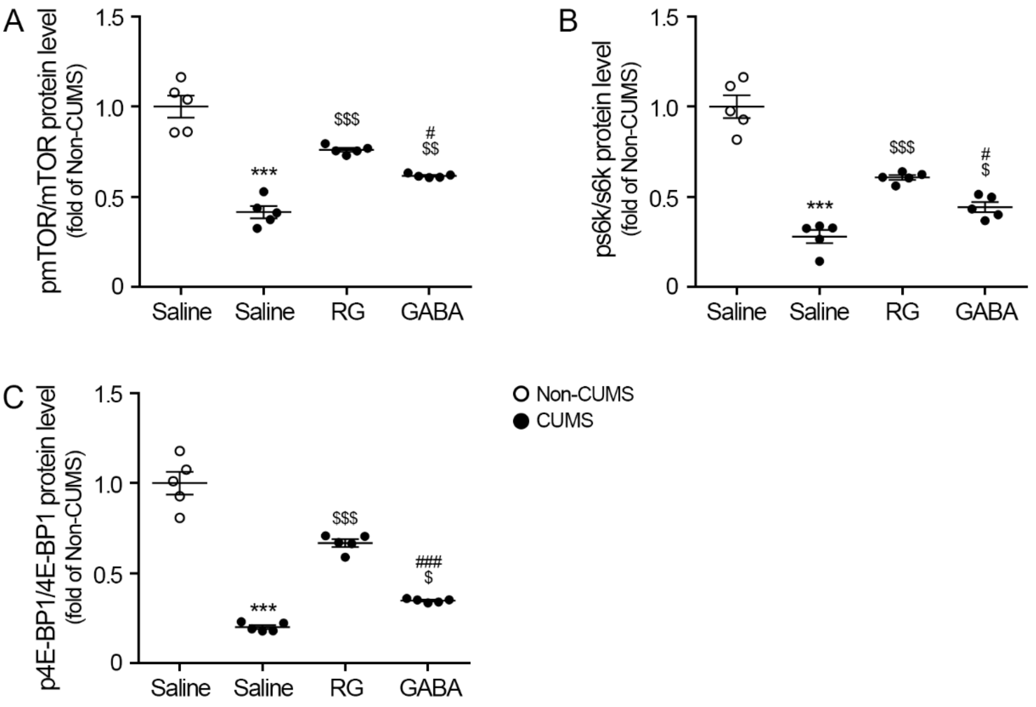

Figure S4. Rice germ (RG) increases the pmTOR/mTOR, ps6k/s6k, and p4E-BP/4E-BP ratios in the gastrocnemius muscle of CUMS mice. (**A–C**) pmTOR/mTOR (A), ps6k/s6k (B), and p4E-BP1/4E-BP1 (C) levels in the gastrocnemius muscle of CUMS mice were analyzed via Western blotting. Graph quantifying the data in Figure 2D. To account for variations in protein loading, the Western blotting quantification was adjusted by normalizing it with β-actin, which served as a control protein for loading. Data are represented as the mean ± standard error of five independent experiments (Sample size, $n = 5$). ***, $p < 0.001$, Non-CUMS/Saline vs. CUMS/Saline; $, $p < 0.05$, $$, $p < 0.01$ or $$$, $p < 0.001$, CUMS/Saline vs. CUMS/RG or GABA; #, $p < 0.05$ or ###, $p < 0.001$ CUMS/RG vs. CUMS/GABA (Tukey's test).

Table S1. List of antibodies Western blotting, ELISA and IHC.

Antibody	Company	Dilution rate ELISA	Dilution rate WB	Dilution rate IHC
ACTH	Santa Cruz	1:200		
Cortisol	Mybiosource	1:2000		
GR	GeneTex	1:1000	1:1000	
pmTOR	Abbexa		1:1000	
mTOR	Sigma-Aldrich		1:1000	
pS6K	Cell Signaling Technology		1:1000	
S6K	Cell Signaling Technology		1:1000	
p4E-BP	Cell Signaling Technology		1:1000	
4E-BP	Cell Signaling Technology		1:1000	
IGF-1	Abcam		1:1000	
pAKT	Abcam		1:1000	
AKT	BD		1:1000	
pFoxO3a	Cell signaling Technology		1:1000	
Foxo3a	ProSci		1:1000	
iNOS	Cell signaling		1:1000	
p53	Santa Cruz		1:1000	
ace-p53	Cell signaling		1:1000	
CDK-2	Abclonal		1:1000	
Cyclin D1	BioLegend		1:1000	
β-actin	Cell signaling		1:1000	
PCNA	Abcam			1:500

Table S2. List of primers of qRT-PCR.

Gene		Primers
Actb	Forward	5'-CCGTAAAGACCTCTATGCCAAC-3′
	Reverse	5'-GCAGTAATCTCCTTCTGCATCC-3′
Klf15	Forward	5'-TTC TGC TTC CCT GAA TTT CTG T-3′
	Reverse	5'-TTC TTC AAT CTC CTC CAG GGT A-3′
Redd-1	Forward	5'-GGC TGT TAA GTT CTG CCA ACT C-3′
	Reverse	5'-GAT GAC TCT GAA GCC GGT ACT T-3′
Atrogin-1	Forward	5'-TGA CAA AGG GCA GCT GGA TT-3′
	Reverse	5'-AGG GTT TCT CAA TGA CAG CG-3′
Murf1	Forward	5'-GTG TGA GGT GCC TAC TTG CT-3′
	Reverse	5'-GAC TTT TCC AGC TGC TCC CT-3′

Table S3. RG contained 30% of GABA. This is an high performance liquid chromatography (HPLC) result analyzing the active substances (e.g. GABA, lactic acid..) contained in the RG.

<table>
<tr><th>Category</th><th>Unit</th><th>Specification</th><th colspan="2">Result</th><th>Verdict</th></tr>
<tr><td>Appearance</td><td></td><td>Pale Yellow Powder</td><td colspan="2">Pale Yellow Powder</td><td>Valid</td></tr>
<tr><td>Foreign Matter</td><td></td><td>Non-detected</td><td colspan="2">Non-detected</td><td>Valid</td></tr>
<tr><td>Moisture</td><td>%</td><td>Below 8.0 %</td><td colspan="2">4.46</td><td>Valid</td></tr>
<tr><td>Gamma Aminobutyric acid</td><td>mg/g</td><td>Above 280</td><td colspan="2">292.2</td><td>Valid</td></tr>
<tr><td rowspan="2">Organic acid</td><td rowspan="2">mg/g</td><td rowspan="2">Below 15</td><td>Lactic acid</td><td>11.9</td><td rowspan="2">Valid</td></tr>
<tr><td>Acetic acid</td><td>1.3</td></tr>
<tr><td>Coliform</td><td>CFU/g</td><td>n=5, c=1, m=0, M=10</td><td colspan="2">0,0,0,0,0</td><td>Valid</td></tr>
<tr><td>Escherichia coli</td><td>CFU/g</td><td>n=5, c=2, m=0, M=10</td><td colspan="2">0,0,0,0,0</td><td>Valid</td></tr>
</table>
